# ASSOCIATION OF BLOOD TYPE AND MORTALITY OF COVID-19: A HOSPITAL-BASED STUDY IN NATIONAL REFERRAL HOSPITAL, INDONESIA

**DOI:** 10.21010/Ajidv17i2.4

**Published:** 2023-03-29

**Authors:** USMAN Elly, KATAR Yusticia

**Affiliations:** 1 Department of Pharmacology, Faculty of Medicine, Universitas Andalas, Padang, Indonesia

**Keywords:** Blood type, COVID-19, Hospitalized, Mortality

## Abstract

**Background:**

The ABO blood type is crucial in a number of illnesses, including cancer, cardiovascular disease, and some communicable and non-communicable illnesses. However, there is currently little clear evidence between COVID-19 with ABO blood types. This study was, therefore, aimed to assess the association between ABO blood type and the mortality of patients infected with COVID-19 in a national referral hospital in Indonesia.

**Materials and Methods:**

This study used a retrospective cohort design. The research sample was COVID-19 patients who were in Dr. M. Djamil Hospital Padang. The number of samples in this study was 93 subjects. The Chi-square test was used in the data analysis. The data were analyzed using the SPSS version 21.0 program, and p<0.05 was considered significant.

**Results:**

The results of this study found the percentage of mortality of COVID-19 patients was higher for blood group O (46.2%), followed by AB (41.7%), B (26.3%), and A (13.9%). There was a relationship between blood type and mortality in hospitalized COVID-19 patients (p <0.05), where blood type O had the highest risk (OR = 5.31, 95% CI 1.57-17.98) followed by blood type AB (OR = 4.43, 95% CI 1.01-19.58).

**Conclusion:**

This study confirmed there was a relationship between blood type and mortality in hospitalized COVID-19 patients, where blood type O had the highest risk followed by blood type AB.

## Introduction

COVID-19 pandemic has taken a devastating impact on the world, regardless of age or gender, but especially on older persons who have co-morbid conditions including diabetes and cardiovascular and cerebrovascular diseases (Almazeedi *et al.*, 2020; Velavan *et al.*, 2020). Indonesia has also faced a significant increase in COVID-19 patients which has resulted in morbidity and even death (Hikmawati *et al.*, 2021).

ABO blood type has been linked to the presence of COVID-19 infection in earlier studies. The ABO blood type is crucial in a number of illnesses, including cancer, cardiovascular disease, and some communicable and non-communicable illnesses (Fan *et al.*, 2020). Several studies have shown that having blood type O significantly lowers the chance of contracting hepatitis B (Mohammadali *et al.*, 2014; Belali, 2022). In addition, people with blood type A are more likely to develop rotavirus gastroenteritis than patients with blood type B (Mohammadali *et al.*, 2014; Elnady *et al.*, 2017). In addition, it was found that those with blood type AB are 2.5 times more likely than people with other blood types to contract dengue fever (Murugananthan *et al.*, 2018). Previous research on SARS-CoV-1 has found a correlation between blood type and the risk of infection, with people with group O blood having a small chance of SARS-CoV-1 infection (Cheng *et al.*, 2005).

There has not been much conclusive evidence to date between SARS-CoV-2 and ABO blood types (Zhao *et al.*, 2021). According to the majority of research, blood type O has a lower probability of developing severe SARS-CoV-2 infection than blood type A, which has a higher risk of infection (Goker *et al.*, 2020). In addition, very little research has been conducted in Indonesia to find a relationship between blood type and mortality of COVID-19 patients (Liu *et al.*, 2021). However, there is increased interest in the relationship between blood type, and the risk of infection. Everyone agrees that individuals differ from one another, but it is rare for people to be able to predict whether a person’s blood type makeup makes them more or less likely to contract an infection when exposed to viruses (Kim *et al.*, 2021). The purpose of this study was to assess the mortality of COVID-19 infected patients with different ABO blood types.

## Materials and Methods

### Study design and research sample

This study used a retrospective cohort. The research sample was COVID-19 patients who were in Dr. M. Djamil Hospital Padang, Indonesia. The research was conducted from January to November 2022. There were 93 total samples in this study. Convenience sampling was used as the sampling method in this study. Patients with COVID-19 with moderate to severe clinical severity and thoroughly documented information related to research variables met the inclusion criteria for this study.

### Operational definition

Blood type was the study’s independent variable (A, B, AB, and O) (Rana *et al.*, 2021). The dependent variable was COVID-19 patient mortality (death, and life) (Sjögren *et al.*, 2021).

### Research ethics approval

The Dr. M. Djamil Hospital Padang research ethics committee approved this study after conducting an ethical review (LB.02.02/5.7/192/2022).

## Data analysis

Research variables in categorical research are provided as frequency and percentage. While numeric variables are presented in the form of mean ± SD or median (min-max). The Mann-Whitney test and the Chi-square test were used in the bivariate analysis. The data were analyzed using the SPSS version 21.0 program, and a p-value< 0.05 was considered significant.

## Results

Table 1 shows subject characteristics.

**Table 1:** Subject characteristics.

Characteristics	Mortality		p-value

	Death (n=27)	Life (n=66)	
**Sex, f(%)**			0.226^a^
Male	21 (33.9)	41 (66.1)	
Female	6 (19.4)	25 (80.6)	
**Age (years), median (min-max)**	59 (21-79)	56 (20-83)	0.249^b^
**BMI (kg/m^2^), median (min-max)**	25.15 (15.82-76.10)	24.80 (17.96-55.51)	0.691^b^
**Clinical severity, f(%)**			<0.001^a*^
Moderate	1 (2.0)	49 (98.0)	
Severe	22 (61.1)	14 (38.9)	
Critical	4 (57.1)	3 (42.9)	
**Laboratory examination, median (min-max)**			
Hb (gr/dL)	10.95 (4.4-17.4)	12.00 (4.0-15.7)	0.482^b^
Ht (%)	28.50 (6-53)	36.00 (15-8,150)	0.114^b^
Leukocytes (/mm^3^)	2,240.50 (66-47,450)	1,400.00 (7-23,160)	0.365^b^
Platelets	191,000 (229-652,000)	194,000 (44-650,000)	0.722^b^
D-Dimer (ng/mL)	4,435.00 (1,039-10,000)	777.00 (157-10,000)	<0.001^b*^
Procalcitonin (ng/mL)	0.74 (0.05-171)	0.27 (0.05-171.42)	0.092^b^
IL-6 (pg/mL)	262.50 (12.20-4,599.00)	34.40 (1.50-600.00)	<0.001^b*^
Ferritin (ng/mL)	1,179.58 (12.40-2,000.00)	710.00 (27.09-2,000.00)	<0.021^b*^
SpO2 (%)	90.00 (48-99)	96.00 (33-99)	<0.001^b*^
**Number of comorbid, f(%)**			0.009^a*^
None	3 (14.3)	18 (85.7)	
1	2 (10.5)	17 (89.5)	
> 1	22 (41.5)	31 (58.5)	

* p<0.05 considered significant; a, Chi-square test; b, Mann-whitney test

Table 1 (above) shows IL-6 levels, ferritin, SpO2 values, PCT levels, D-Dimer, clinical severity, and the number of comorbidities associated with mortality in COVID-19 patients hospitalized (p<0.05).

**Figure 1:**
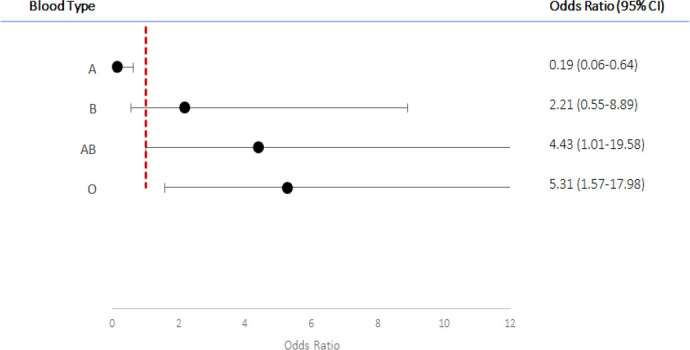
Forest plot blood type and mortality of COVID-19.

The association of blood type and mortality of COVID-19 are presented in Table 2 and Figure 1.

**Table 2:** Association of blood type and mortality of COVID-19.

Obese	Mortality		p-value	OR (95% CI)

	Death (f/%) (n=27)	Life (f/%) (n=66)		
A	5 (13.9)	31 (86.1)	0.543^a^	0.19 (0.06-0.64)
B	5 (26.3)	14 (73.7)	0.054^a^	2.21 (0.55-8.89)
AB	5 (41.7)	7 (58.3)	0.021*^a^	4.43 (1.01-19.58)
O	12 (46.2)	14 (53.8)	0.009*^a^	5.31 (1.57-17.98

* p<0.05 considered significant; a, Chi-square test

Table 2 shows the percentage of deaths of COVID-19 patients was higher for blood group O (46.2%), followed by AB (41.7%), B (26.3%), and A (13.9%). There was a relationship between blood type and mortality in hospitalized COVID-19 patients (p <0.05), where blood type O had the highest risk (OR = 5.31, 95% CI 1.57-17.98) followed by blood type AB (OR = 4.43, 95% CI 1.01-19.58).

## Discussion

The results of this study indicate that the percentage mortality of COVID-19 patients was significantly higher for blood group O (46.2%), followed by blood group AB (41.7%), blood group B (26.3%) and blood group A (13.9%), respectively. There was a relationship between blood type and mortality in hospitalized COVID-19 patients, where blood type O had the highest risk (OR = 5.31) followed by blood type AB (OR = 4.43).

A previous study has stated that blood type is not associated with the risk of intubation or death in COVID-19 patients. Patients with blood types B and AB are more at risk of experiencing COVID-19 infection (Latz *et al.*, 2020). According to another study, COVID-19 patients had higher prevalence of blood types A and AB. Patients with blood categories A and AB reported delayed seroconversion despite the fact that ABO blood type was not related to presentation or recovery times for COVID-19 (Mahmud *et al.*, 2021).

Apart from anti-A antibodies, the link between group A and severe COVID-19 is increased activity of angiotensin-converting enzyme 1 (ACE-1), with a tendency for cardiovascular complications (Gasso *et al.*, 2014). The severe outcome could also be explained by the higher levels of von Willebrand Factor (vWF) and factor VIII in group A individuals. This increases the risk of thromboembolic disease and the severity of COVID-19 which can lead to death (Dai, 2020).

An individual’s blood has a unique characteristic known as a blood type. Because the red blood cell membrane’s surface contains various types of proteins and carbohydrates (Cooling, 2015). Through a variety of processes, blood group antigens can affect the likelihood of developing a disease. These include acting as immune system modifiers in the form of anti-ABO antibodies and acting as immune system receptors or shams for pathogenic pathogens (Leaf *et al.*, 2020).

Previous studies identified inconsistent results, but it did discover certain tendencies that might point to a blood type-related greater vulnerability to COVID-19 infection or to a blood type-related protective effect for certain Rh-negative blood types (Miotto *et al.*, 2021; Rana *et al.*, 2021). COVID-19 is not only at risk by blood type but many other factors, including age or pre-existing medical disorders, are likely to have a larger, dominant role in defining personal risk from COVID-19, regardless of whether a particular blood type is linked to a higher risk of catching COVID-19 and developing severe disease (Djanas *et al.*, 2021; Pendu *et al.*, 2021).

The strength of this research is that it is the first study conducted to examine the relationship between blood type and death in COVID-19 patients treated in an Indonesian hospital. The limitations of this study were that the use of a retrospective cohort study in this study was not fully able to minimize bias, so a cohort study was needed to explain the causality that occurred. Apart from that, because this study only involved one referral hospital in Indonesia, further research is needed on several referral hospitals in Indonesia, so that the results can be representative of the population. In another study, COVID-19 patients had higher prevalences of blood types A and AB. Patients with blood categories A and AB reported delayed seroconversion despite the fact that ABO blood type was not related to presentation or recovery times for COVID-19.

The implication of this study according to the study’s findings, COVID-19 patients who have blood types O and AB are more likely to die or suffer other complications. Furthermore, more research is required to comprehend the molecular pathways through which blood groups may influence vulnerability to COVID-19 infection and, ultimately, to create treatments for viral infections and illnesses. Prioritizing patient treatment should be done by healthcare professionals based on the authors’ advice to blood type patients with COVID-19 before treating them.

## Conclusion

This study identified the relationship between blood type and death in COVID-19 patients who were hospitalized, where blood type O had the highest risk followed by blood type AB. This study can help with the therapeutic management of COVID-19 patients based on blood type so as to reduce the poor prognosis.
